# Whole-Genome Sequence of the Ancestral Animal-Borne ST398 *Staphylococcus aureus* Strain S123

**DOI:** 10.1128/genomeA.00692-13

**Published:** 2013-08-29

**Authors:** Nathalie van der Mee-Marquet, David Hernandez, Xavier Bertrand, Roland Quentin, Anna-Rita Corvaglia, Patrice François

**Affiliations:** Service de Bactériologie et Hygiène, Centre Hospitalier Régional Universitaire, Tours, Francea; Réseau des Hygiénistes du Centre, Centre Hospitalier Universitaire, Tours, Franceb; UMR 6249 Chrono-environnement, Université de Franche-Comté, Besançon, Franced; Genomic Research Laboratory, University of Geneva Hospitals, Geneva, Switzerlandc

## Abstract

Sequence type 398 (ST398) *Staphylococcus aureus* was originally reported in livestock. We announce the complete genome sequence of an ST398 methicillin-susceptible *S. aureus* strain of animal origin, S123. The genome sequence reveals a wild-type genome that probably corresponds to an ancestral clone*.*

## GENOME ANNOUNCEMENT

Sequence type 398 (ST398) *Staphylococcus aureus* was originally described in livestock (1) but was recently reported as the causative agent of severe infections in humans (2). An evolutionary hypothesis suggests that an ancestral wild-type *S. aureus* strain acquired mobile genetic elements triggering host adaptation and evolution to the methicillin-resistant genotype after insertion of a staphylococcal cassette chromosome *mec* (SCC*mec*) element (3). We report here the genome sequence of a carriage methicillin-susceptible *S. aureus* isolate showing sequence type 398 and devoid of any phage element.

*S. aureus* strain S123 was recovered from a pig during a screening campaign in a Dutch farming area in 2010. Purified genomic DNA was subjected to whole-genome shotgun sequencing by using an HiSeq2000 system (Illumina, Inc.). Following fragmentation, end reparation, and sample tagging, the sequencer produced 21.6 million 100-bp paired-end reads, yielding appreciable coverage of around 800×. Assembly was performed using Edena 3.0 (4) and resulted in 49 contigs. The larger contigs showed sizes of 498,000 bp. Overall assembly values were satisfactory, showing a total length of 2.70 Mbp and an *N*_50_ of 152,000 bp. A total of 2,475 predicted coding sequences (CDS) were detected by rapid annotations using subsystems technology (RAST) (5). More than 55% of the genes were assigned to specific subsystem categories by RAST (5). In addition to CDS, RAST revealed 77 structural genes, including 59 tRNA and 18 rRNA genes. Note that S123 contains two circular plasmids of 15 kb and 4.5 kb at apparent copy numbers of 2 and 10, respectively.

The genome of strain S123 encodes known virulence factors, such as pore-forming toxins (only hemolysins) and bacterial adhesins. Annotation confirmed the absence of important resistance determinant genes, except those for tetracycline and erythromycin. Interestingly, strain S123 is missing a type IV restriction system. Similar to many other sequenced ST398 strains, S123 is devoid of DNA from phagic elements despite the presence of a functional type I restriction system.

We conclude that S123 is potentially an ancestral clone still missing a phagic element that contributes to host adaptation.

### Nucleotide sequence accession number.

The whole-genome sequence of *S. aureus* S123 was deposited in the DDBJ/EMBL/GenBank databases under the accession number AUPU00000000.
